# The combination of venetoclax with dimethyl fumarate synergistically induces apoptosis in AML cells by disrupting mitochondrial integrity through ROS accumulation

**DOI:** 10.1038/s41419-025-08040-x

**Published:** 2025-10-21

**Authors:** Shin-ichiro Kawaguchi, Kazuya Sato, Junko Izawa, Norihito Takayama, Hiroko Hayakawa, Kaoru Tominaga, Hitoshi Endo, Tom Kouki, Nobuhiko Ohno, Yoshinobu Kanda

**Affiliations:** 1 Division of Hematology, Department of Medicine, Jichi Medical University, Tochigi, Japan; 2 Center for Cytometry Research, Jichi Medical University, Tochigi, Japan; 3 Division of Structural Biochemistry, Department of Biochemistry, Jichi Medical University, Tochigi, Japan; 4 Division of Functional Biochemistry, Department of Biochemistry, Jichi Medical University, Tochigi, Japan; 5 Division of Histology and Cell Biology, Department of Anatomy, Jichi Medical University, Tochigi, Japan

**Keywords:** Acute myeloid leukaemia, Clinical pharmacology

## Abstract

Leukemia cells are consistently subjected to higher oxidative stress than normal cells. To mitigate reactive oxygen species (ROS) overload, which can trigger various forms of cell death, leukemia cells employ a robust antioxidant defense system and maintain redox homeostasis. Recent evidence suggests that dimethyl fumarate (DMF), a derivative of fumarate, inactivates the antioxidant glutathione (GSH), thereby inducing oxidative stress and metabolic dysfunction, eventually leading to cell death in cancer cells. In this study, we observed that DMF decreases the GSH/oxidated GSH ratio and increases intracellular ROS levels, the extent of which is closely correlated with cell death, in acute myeloid leukemia (AML) cell lines. DMF reduced the mitochondrial membrane potential and oxidative phosphorylation (OXPHOS), effects that were almost fully restored by the antioxidant N-acetylcysteine, suggesting that these responses are ROS-dependent. Electron microscopy and inhibition assays revealed that apoptosis, rather than necroptosis or ferroptosis, is the predominant form of cell death of AML cells following DMF treatment. Notably, the combination of DMF and the BCL-2 selective BH3-mimetic venetoclax induced marked cell death in AML cells, including venetoclax-refractory　BCL-2 low expressing U937 and acquired venetoclax-resistant MOLM-14 cells. This combination also caused greater mitochondrial depolarization and a more profound reduction in OXPHOS activity than either agent alone. Collectively, our findings indicate that DMF exerts potent anti-leukemia activity in AML cells and sensitizes cells to venetoclax treatment by synergistically disrupting mitochondrial integrity through ROS accumulation.

## Introduction

Cancer cells enhance their metabolism and develop unique metabolic pathways distinct from those in normal cells. Enhanced metabolic activity leads to the generation of excessive reactive oxygen species (ROS). While a small amount of ROS is essential for cell homeostatic signaling, excessive ROS accumulation can cause oxidation of cellular structures, including nucleic acids, lipids, and proteins, thereby disrupting mitochondrial dysfunction. This compromises membrane integrity, sensitizes the cells to cytotoxic stimuli, and eventually leads to cell death [1–3]. To counteract this, cancer cells have developed highly sophisticated antioxidant systems, including the upregulation of antioxidant enzymes like glutathione (GSH) peroxidase/reductase and superoxide dismutase. Recent researches have focused on targeting ROS and these antioxidant machineries in cancer cells to induce oxidative stress and promote cell death [2, 4].

Dimethyl fumarate (DMF), a derivative of fumarate, has been approved globally for the treatment of relapsing multiple sclerosis and psoriasis [5, 6]. While the precise mechanism of action is not fully elucidated, post-translational modification of proteins through succination of cysteine residues has been considered to play a major role in its functions. Among the various potential target molecules is GSH. Loss of GSH activity can cause intracellular accumulation of ROS, leading to apoptosis in diverse immune cells and cancers [7–10]. So far, the anti-tumor effects of DMF have been extensively investigated in various cancers and lymphomas, providing encouraging results for future clinical applications. On the contrary, however, there are only few reports on myeloid malignancies, and no consensus has yet been reached regarding its therapeutic potential [11, 12].

In this study, we demonstrate that DMF reduces GSH levels and promotes ROS accumulation, leading to apoptosis in AML cell lines, and the extent of these effects varies among different cell lines. Increased ROS levels are significantly correlated with reduced cell viability and are also associated with a decrease in mitochondrial membrane potential (MMP) and oxidative phosphorylation (OXPHOS). Moreover, we observed significant synergy between the BH3-mimetic venetoclax and DMF, as this combination promoted higher ROS accumulation and reduced MMP, inducing more apoptosis in AML cell lines than either agent alone. Our findings support the therapeutic strategy of targeting GSH activity in AML cells, which leads to ROS generation and impairs mitochondrial integrity, further sensitizing the cells to the BH3-mimetic venetoclax.

## Methods

### Cell lines

Human AML cell lines (MOLM-14, U937, KG-1a and THP-1) were obtained from Japanese Collection of Research Bioresources Cell Bank. Cells were cultured and maintained in RPMI-1640 medium supplemented with 10% fetal bovine serum, 2mM L-glutamine, 0.1 mg/mL streptomycin, and 100 U/mL penicillin G. Generation of venetoclax-resistant cell lines has been described elsewhere [11]. Finally, we generated venetoclax-resistant MOLM-14 cells that were stably viable in medium containing 4 µM venetoclax.

### CRISPR/Cas9 genome editing

The Nrf2 gene was knocked out by CRISPR/Cas9 technology using a lentiviral vector coding for the single-guide RNA hCas9 and puromycin resistance (VectorBuilder, pLV[CRISPR]-hCas9/Puro-U6 > hNFE2L2[gRNA#3806]). Scramble gRNA lentiviral vector with no known target in the human genome was used as a reference (VectorBuilder).

### Flow cytometry

Cells were stained in HBSS supplemented with 2% bovine serum albumin, 1 mM EDTA and 25 mM HEPES with a combination of antibodies listed in Supplementary Table S1. To identify apoptotic cells, cells were stained with PE Annexin V in Annexin V Binding Buffer (BioLegend) for 20 minutes at room temperature, followed by 7-AAD staining. For MitoSox Red staining and MitoTracker™ Red CMXRos in combination with MitoTracker™ Green FM staining, cells were incubated in Dulbecco’s PBS without calcium or magnesium and Phenol Red-free RPMI1640 (Thermo Fisher), respectively. All events were acquired on a BD LSRFortessa (BD Biosciences), and subsequent analysis was conducted using FlowJo software (FlowJo LLC).

### Cell proliferation assay

Cells were stained using a Premix WST-1 Cell Proliferation Assay System (Takara Bio Inc., Shiga, Japan). The microplate was read by measuring the absorption at 440 nm using a spectrophotometer. Combination effects and synergy scores were analyzed using the SynergyFinder web application (https://synergyfinder.org/) [12].

### Measurement of oxygen consumption rate

Oxygen consumption rate (OCR), an indicator of OXPHOS, was measured using the Seahorse XF Cell Mito Stress Test Kit (Seahorse Bioscience). With the use of a Seahorse XF96 extracellular flux analyzer, OCR was measured under basal conditions followed by sequential treatment with oligomycin (an ATP synthase inhibitor, 1 μM), carbonylcyanide p- trifluoromethoxyphenylhydrazone (FCCP, an uncoupling agent, 1 μM), and a combination of rotenone (complex I inhibitor, 0.5 μM) and antimycin A (complex III inhibitor, 0.5 μM).

### Colony-forming unit assay

Frozen human bone marrow-derived CD34^+^ cells were obtained from STEMCELL technologies (Vancouver, BC, Canada). A total of 10,000 viable cells were suspended in 300 μL of the Cell Resuspension Solution, mixed with 3 mL of Human Methylcellulose Complete Media (HSC003, R&D Systems, Minneapolis, MN), and plated into a 3-cm tissue culture dish. Colony formation was assessed 10–14 days after initial plating.

### Measurement of GSH and oxidized glutathione

Cell extracts were prepared by sonication in ice-cold 5% metaphosphoric acid and the supernatant fluid was collected after centrifugation. GSH content and the ratio of total reduced to oxidized glutathione (GSSG) were determined by a GSSG/GSH quantification kit (Dojindo Laboratories). GSSG was detected by measuring the absorption of Ellman’s reagent using a spectrophotometer at 405 nm. GSH was determined by subtracting GSSG from the total amount of glutathione.

### Western blot analysis

Cells were lysed with M-PER mammalian protein extraction reagent (Thermo Fisher) supplemented with cOmplete protease inhibitor and PhosSTOP phosphatase inhibitor cocktails (Roche). Nuclear and cytoplasmic protein were fractionated using the LysoPure^TM^ Nuclear and Cytoplasmic Extractor Kit (Wako). Samples were separated using polyacrylamide gels and transferred using the iBlot dry transfer system (Thermo Fisher). The membranes were blocked with 5% skim milk in TBST and then incubated with primary antibodies (Supplementary Table S2) overnight at 4 °C, followed by incubation with the corresponding HRP-conjugated secondary antibody for 1 h at room temperature. The blots were imaged on the GeneGnome chemiluminescence imaging system (Syngene International Ltd.) after incubation with SuperSignal™ West Pico PLUS Chemiluminescent Substrate (Thermo Fisher).

### Fluorescence and transmission electron microscopy

Wright-Giemsa-stained cytospin preparations of leukemia cells were observed under an Olympus BX63 microscope, and images were captured using a DP80 camera (Olympus). Transmission electron microscopy was performed as described previously [13].

### Statistical analysis

One-way ANOVA, Tukey’s post-hoc comparisons and calculation of the Pearson product-moment correlation coefficient were performed by Prism 10.1.0 (GraphPad Software) or EZR (Saitama Medical Center, Jichi Medical University), a graphical user interface for R (The R Foundation for Statistical Computing) [14]. More precisely, it is a modified version of R Commander designed to add statistical functions frequently used in biostatistics. All tests were two-sided.

## Results

### DMF induces ROS accumulation and promotes cell death of AML cells in a dose-dependent manner

**Fig. 1:**
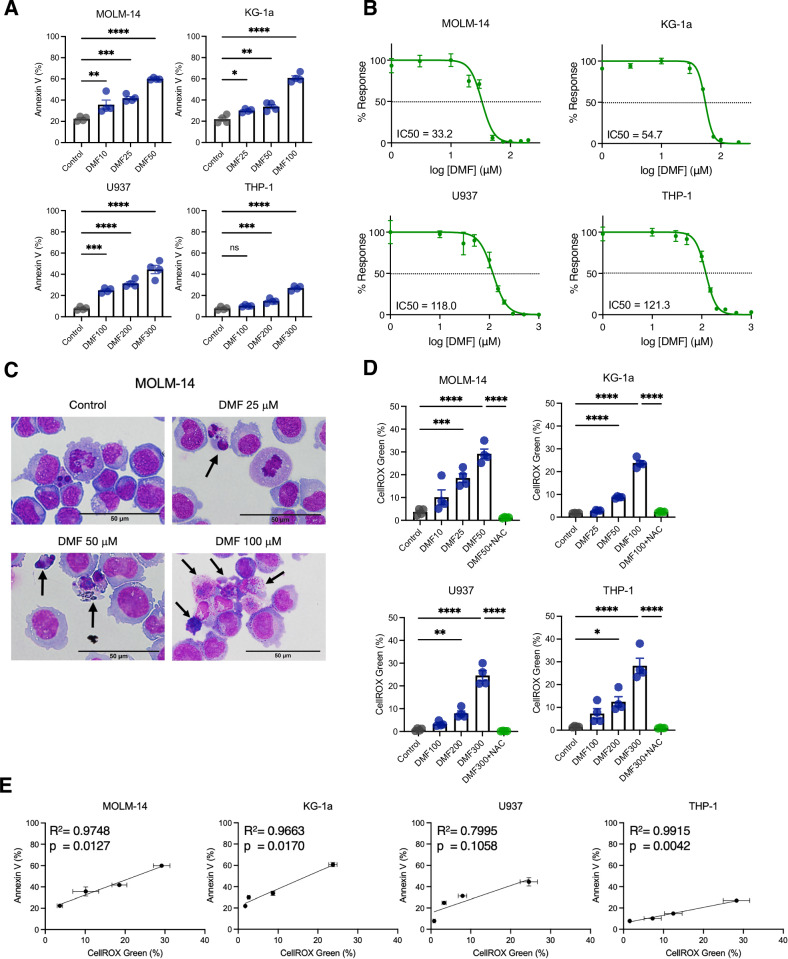
DMF induces ROS accumulation and promotes cell death in human AML cell lines in a dose-dependent manner. Cell viability was assessed in human AML cell lines (MOLM-14, U937, KG-1a, and THP-1) following DMF treatment. Cells (2 × 10^5^ cells/well in a 12-well plate) were treated in the presence or absence of DMF. The time points of analysis were 24 h (Annexin V, WST-1 assay) and 6 h (Cellular ROS). **A** Bar graphs showing Annexin V-positive cells (%) measured by flow cytometry. * *P* < 0.05, ** *P* < 0.01, *** *P* < 0.001, **** *P* < 0.0001, ns: not significant, one-way ANOVA with Tukey’s post-hoc test. Shown as mean ± SEM (*n* = 4 per group). Bars show mean values ± SEM from one of three independent experiments. **B** Response of AML cell lines to various concentrations of DMF. The cell viability values obtained from treated cells were normalized with respect to the untreated samples (*n* = 4-5 per group). The half-maximal inhibitory concentration (IC_50_) was calculated by Prism software. **C** Representative optical microscopical images of MOLM-14 cells (40x). **D** ROS levels in AML cell lines treated with increasing concentrations of DMF. Cellular ROS levels were measured using CellROX Green oxidative stress probe. N-acetylcysteine (NAC; 2.5 mM) was used concomitantly with DMF at the highest concentrations in each cell line. * *P* < 0.05, ** *P* < 0.01, *** *P* < 0.001, **** *P* < 0.0001, one-way ANOVA was used and adjusted for multiple comparisons. Shown as mean ± SEM (*n* = 4 per group). Bars show mean values ± SEM from one of at least three independent experiments. **E** Correlation between ROS levels and Annexin V positivity in AML cell lines treated with DMF. ROS levels were determined by CellROX green (%) in cells 6 h following DMF treatment, whereas cell viability was assessed at 24 h. Linear modeling of the Pearson product-moment correlation coefficient was used to score the correlations.

We first investigated the anti-leukemic effect of DMF on four genetically and biologically distinct AML cell lines, namely MOLM-14, U937, KG-1a and THP-1 (Supplementary Table S3). Flow cytometry analysis showed that DMF induced a significant increase in cell death in all cell lines, in a dose-dependent manner (Fig. 1A). The WST-1 cell proliferation assay showed a similar trend (Fig. 1B). MOLM-14 and KG-1a were highly susceptible to DMF (IC_50_ = 33.2 µM and 54.7 µM, respectively), whereas U937 and THP-1 required relatively higher concentrations to induce cell death (IC_50_ = 118.0 µM and 121.3 µM, respectively). Morphologically, a greater number of cells underwent nuclear fragmentation and aggregation as the concentration of DMF increased (Fig. 1C**and** Supplementary Fig. S1A).

Under oxidative conditions, intracellular GSH reacts with hydrogen peroxide, and is converted into the oxidized form, GSSG. It has been shown that fumarate and its derivatives are capable of succinating several cysteine-rich peptides, including GSH. Succinated GSH (GSF) loses its reducing function and inhibits GSH generation [7]. Several reports have demonstrated that DMF induces oxidative stress by depleting GSH activity and promotes cell death in solid tumors and lymphomas [15–17]. Therefore, we investigated whether, and if so how, DMF influences the redox status and cell viability in AML cells.

Following a six-hour treatment with DMF, intracellular ROS levels determined by CellROX Green were elevated in all AML cell lines in a dose-dependent manner (Fig. 1D). ROS levels in MOLM-14 cells increased within one hour after drug treatment, peaked at 12 h, and maintained even after 24 h (Supplementary Fig. S1B). The antioxidant and GSH precursor substrate NAC reduced ROS to the basal levels. Consequently, a strong correlation was observed between ROS levels and the rate of cell death induced by DMF (Fig. 1E). Likewise, MitoSox fluorescence intensity was increased in MOLM-14 following DMF treatment, suggesting that at least some ROS were derived from mitochondrial superoxide (Supplementary Fig. S1C). Consistent with these observations, Mito-TEMPO, a mitochondria-targeted superoxide dismutase mimetic, significantly reduced mitochondrial ROS levels. Furthermore, DMF treatment resulted in a substantial decrease in the GSH/GSSG ratio in MOLM-14, U937, and KG-1a, whereas it had a comparably modest effect on THP-1 (Supplementary Fig. S1D**, left**). NAC restored the GSH/GSSG ratio (Supplementary Fig. S1D**, right**) and inhibited DMF-induced cell death in all cell lines (Supplementary Fig. S1E), suggesting that ROS play a central role in the anti-leukemia effects of DMF.

### DMF-mediated ROS induce mitochondrial depolarization and reduce OXPHOS followed by apoptosis in AML cell lines

**Fig. 2:**
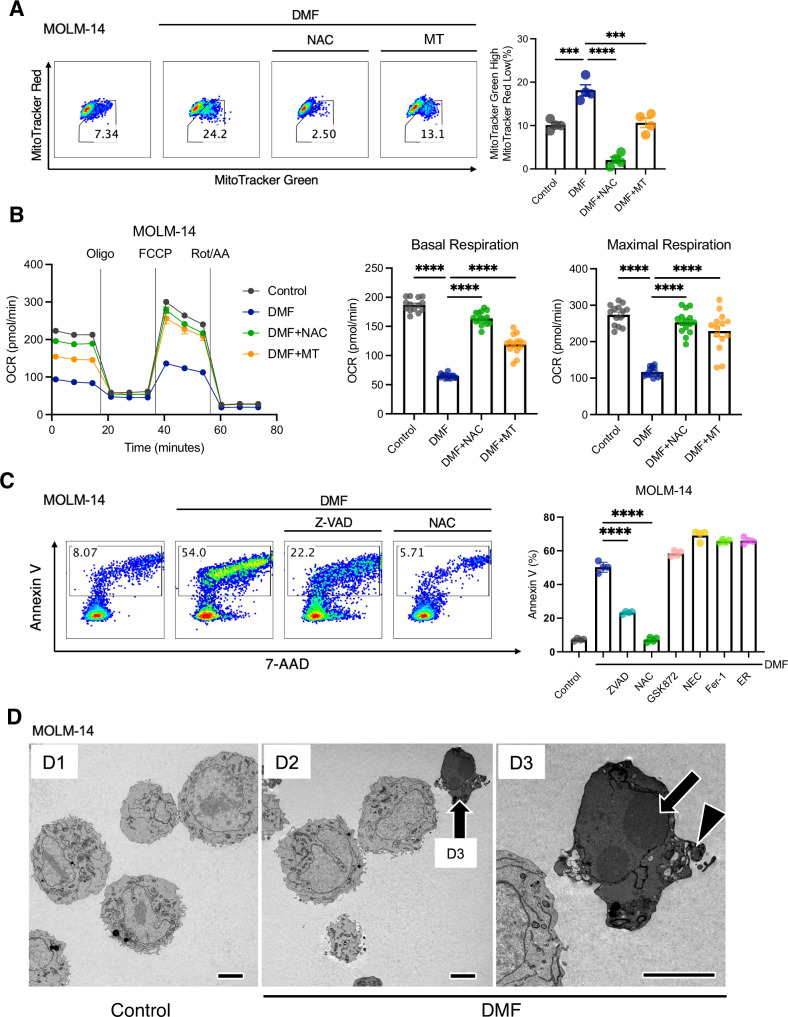
DMF induces mitochondrial depolarization and reduces OXPHOS through ROS accumulation followed by apoptosis in human AML cell lines. **A** Flow cytometric analysis of mitochondrial status on MOLM-14. Scatter plots are representative of three independent experiments (**left**). Bar graphs show the MitoTracker Green high and MitoTracker Red low population (%), which represents depolarized mitochondria (**right**). Cells (2 × 10^5^ cells/well in a 12-well plate) were treated with DMF (50 μM) in the presence or absence of NAC (2.5 mM) or Mito-TEMPO (MT; 5 μM) for 6 h. **B** Seahorse extracellular flux assay. OCR in MOLM-14 was measured after sequential treatment with oligomycin (Oligo), FCCP, and a combination of rotenone/antimycin A (Rot/AA) in MOLM-14. Cells were preincubated with medium containing 0.1% DMSO (control), or DMF (50 µM) in the presence or absence of NAC (2.5 mM) or Mito-Tempo (5 μM) overnight. Basal respiration was measured prior to sequential treatment. Graphs compare the basal and maximal OCR (*n* = 15 per group). **** *P* < 0.0001. one-way ANOVA with Tukey’s post-hoc test. Shown as mean ± SEM. **C** Flow cytometric analysis of apoptosis induced by DMF in the presence or absence of various inhibitors. Cells (2 × 10^5^ cells/well in a 12-well plate) were exposed to medium containing 0.1% DMSO (control), or DMF (50 µM) in the presence or absence of the indicated inhibitors (NAC; 2.5 mM), Z-VAD-fmk (ZVAD; 50 µM), GSK872 (10 μM), Necrostatin-1 (NEC; 30 μM), Ferrostatin-1 (Fer-1; 5 μM), or ER000444793 (ER; 10 μM) for 24 h. **** *P* < 0.0001, one-way ANOVA with Tukey’s post-hoc test. Shown as mean ± SEM (*n* = 4 per group). **D** Representative electron microscopy images of MOLM-14 cells at low (D1: Control, D2: DMF 50 μM) and high (D3: DMF 50 μM) magnifications. Note that under DMF treatment, a few cells have an abnormal electron density (D2, arrow, magnified in D3) along with chromatin condensation (D3, arrow) and cell membrane blebbing (D3, arrowhead). Scale bars 5μm.

ROS can cause the oxidation of mitochondrial structures, which in turn leads to mitochondrial membrane depolarization and dysfunction of the electron transport chain (ETC). To determine viable functional mitochondria in AML cells by flow cytometry, we stained the cells with different mitochondrial dyes: MitoTracker Green, an MMP-insensitive probe, which reports mitochondrial content; and MMP-sensitive MitoTracker Red, which shows functionally viable mitochondria. Flow cytometry analysis demonstrated a significant increase in depolarized mitochondria (MitoTracker Green^high^ and MitoTracker Red^low^) in the DMF-treatment group (Fig. 2A). Both NAC and Mito-TEMPO reduced the percentage of depolarized mitochondria to a basal level. Besides, an extracellular flux analyzer revealed that the OCR, an indicator of mitochondrial OXPHOS, was significantly decreased by DMF treatment (Fig. 2B). This inhibitory response was abrogated by NAC and Mito-Tempo, suggesting that ROS accumulation is not the result of, but rather the cause of impaired mitochondrial respiration. Collectively, these observations suggest that DMF targets mitochondrial biological activity through the overload of cellular ROS in AML cells.

ROS have been shown to mediate four distinct types of programmed cell death in cancer cells: apoptosis, necroptosis, ferroptosis, and opening of the mitochondrial permeability pore (mPTP), which causes the release of cytochrome C from the mitochondria to the cytosol. Thus, we sought to determine the underlying mechanisms by which DMF induces cell death in AML cells through ROS accumulation. Among all the inhibitors we tested, only Z-VAD-fmk, a pan-caspase inhibitor, was able to attenuate DMF-derived cell death in MOLM-14 (Fig. 2C**left**). Other inhibitors, including GSK872 (RIPK3), Necrostatin-1 (RIPK1), Ferrostatin-1 (ferroptosis), and ER000444793 (mPTP opening) did not have similar effects (Fig. 2C**right**). Electron microscopy clearly demonstrated chromatin condensation and cell membrane blebbing in DMF-treated cells, which are hallmark morphological characteristics of apoptotic cell death (Fig. 2D).

### Venetoclax combined with DMF has potent anti-leukemia activity in AML cell lines

**Fig. 3:**
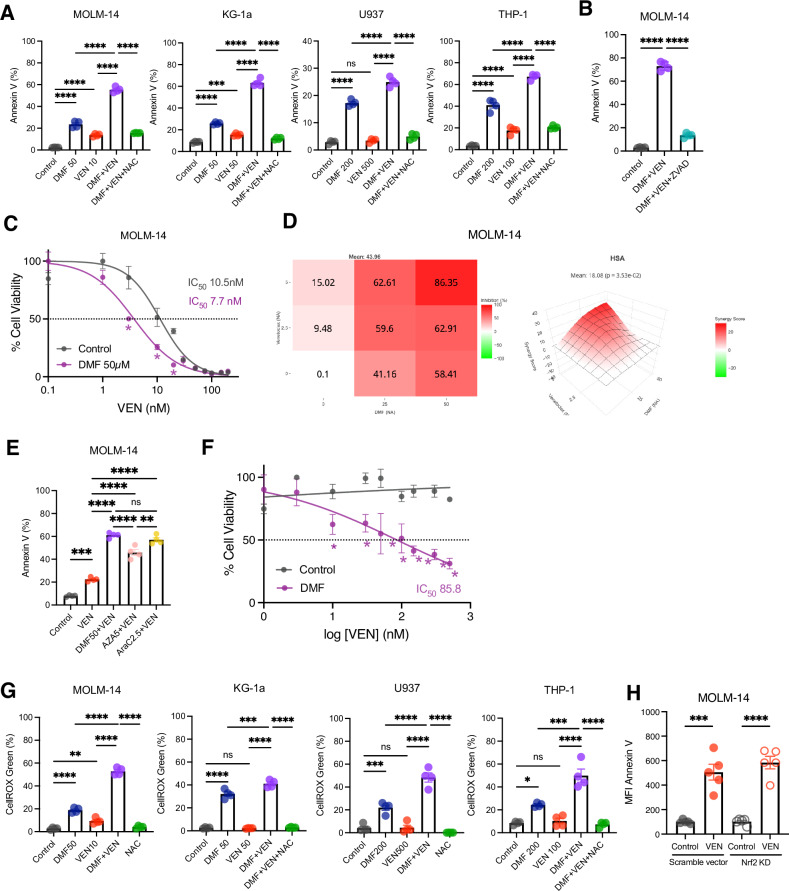
The combination of venetoclax and DMF has potent anti-leukemia activity in AML cell lines. **A, B** Annexin V-positive cells (%) in human AML cell lines. **** *P* < 0.0001, one-way ANOVA with Tukey’s post-hoc test. Human AML cell lines (MOLM-14, U937, KG-1a and THP-1; 2 × 10^5^ cells/well in a 12-well plate) were treated under the indicated conditions for 24 h (*n* = 4 per group). NAC and Z-VAD-fmk were used at 2.5 mM and 50 µM, respectively. The time points of analysis were 24 h (Annexin V, WST-1 assay) and 6 h (Cellular ROS). Data are presented as the mean ± SEM. **C** Dose-response curves for venetoclax in MOLM-14 cells. Cells were treated with varying concentrations of venetoclax with or without DMF (50 µM). Data are shown as the mean ± SEM (*n* = 4-5 per group). * *P* < 0.001, Student t-test. **D** Combination synergy between venetoclax and DMF in MOLM-14 cells. Synergy scores were calculated by the Highest Single Agent (HSA) model using the SynergyFinder web application. **E** Annexin V-positive cells (%) in MOLM-14 cells. Venetoclax, DMF, azacitidine, and cytarabine was used at 10 nM, 50 µM, 5 µM, and 2.5 µM, respectively. ** *P* < 0.01, *** *P* < 0.001, **** *P* < 0.0001, ns: not significant, one-way ANOVA with Tukey’s post-hoc test. **F** Dose-response curves for venetoclax in venetoclax-resistant MOLM-14 cells. Cells were treated with varying concentrations of venetoclax with or without DMF (100 µM). Venetoclax-resistant MOLM-14 cells were generated as described in the **Materials and Methods**. Data are shown as the mean ± SEM (*n* = 4-5 per group). * *P* < 0.001, Student t-test. **G** Cellular ROS levels in AML cell lines. * *P* < 0.05, ** *P* < 0.01, *** *P* < 0.001, **** *P* < 0.0001, ns: not significant, one-way ANOVA with Tukey’s post-hoc test. Shown as mean ± SEM. **H** Comparison of venetoclax sensitivity of wild-type MOLM-14 and Nrf2-knocked down (KD) MOLM-14 cells. Flow cytometric analysis of Annexin V staining between wild-type MOLM-14 and Nrf2 KD MOLM-14 cells following venetoclax treatment (10 nM, 24 h).

Since BCL-2 family proteins have been shown to not only regulate the apoptotic pathway but also to play a central role in mitochondrial metabolism [18, 19], we hypothesized that the combination of the BCL-2 inhibitor venetoclax and DMF could serve as an alternative therapeutic strategy for targeting mitochondrial function and the redox balance in AML. In general, BCL-2 expression levels strongly correlate with the sensitivity of AML cells to venetoclax: MOLM-14 cells are highly dependent on BCL-2 and exhibit significant sensitivity to venetoclax, whereas U937 cells, which show lower BCL-2 expression, are highly refractory to venetoclax treatment [20, 21]. As described in other reports, we observed a significant reduction in the viability of MOLM-14 cells under a low concentration (10 nM) of venetoclax (Fig. 3A). While this effect was further enhanced by combining with DMF (Fig. 3A), viability was significantly restored by Z-VAD-fmk (Fig. 3B), suggesting that this synergy is based on apoptotic cell death. Indeed, apoptotic cells were more frequently observed among MOLM-14 cells treated with this combination than among those treated with either single agent (Supplementary Fig. S3A). Furthermore, we confirmed that the combination of venetoclax and DMF had synergistic effects in MOLM-14 cells using curve-shift and combination matrix analysis (Fig. 3C, D). The extent of apoptosis induced by venetoclax and DMF was comparable to that observed when venetoclax was combined with either azacitidine or cytarabine (Fig. 3E). Notably, the combination of venetoclax and DMF, when applied at the same concentrations, did not impair the colony-forming ability of human normal CD34^+^ cells (Supplementary Fig. S3B).

Conversely, U937 did not undergo cell death by venetoclax, even at 500 nM (Fig. 3A). However, venetoclax combined with DMF induced more cell death in U937 than did DMF alone. Similarly, KG-1a and THP-1 cells, which were moderately susceptible to venetoclax treatment, also exhibited significant apoptosis when combined with DMF. Moreover, DMF also restored the sensitivity to venetoclax in venetoclax-resistant MOLM-14 cells, which had been generated by continuous exposure to increasing concentrations of venetoclax, albeit to a lesser extent than in wild-type MOLM-14 (Fig. 3F). This suggests that this synergy is independent of acquired resistance to subsequent venetoclax treatment in AML.

Although the increase in ROS levels by single-agent venetoclax was modest or even absent when used at low concentrations, the combination of venetoclax and DMF led to significantly higher levels of ROS accumulation in all AML cell lines (Fig. 3G). This suggests that a redox imbalance may trigger this synergistic inhibition. A previous study proposed that venetoclax with hypomethylating agents inhibits the nuclear translocation of antioxidant nuclear factor erythroid 2-related factor 2 (Nrf2) and subsequent antioxidant response in AML cells [22]. Western blot analysis demonstrated that DMF facilitated the nuclear translocation of Nrf2 protein, probably due to a compensatory response to GSH inactivation and subsequent ROS accumulation; however, venetoclax had no effect on Nrf2 activation (Supplementary Fig. S3C). In line with this observation, the expression level of Nrf2 downstream *NQO1* mRNA was not downregulated following venetoclax treatment (Supplementary Fig. S3D). Furthermore, genetic knockdown of Nrf2 by CRISPR/Cas9 genome editing did not affect the susceptibility of MOLM-14 cells to venetoclax (Fig. 3H, Supplementary Fig. S3E and S3F). More recently, another report suggested that the combination of venetoclax and azacitidine reduces cystine uptake, impairing GSH synthesis and subsequent ROS accumulation [23]. However, neither intracellular GSH levels (Supplementary Fig. S3G) nor cystine uptake activity (Supplementary Fig. S3H) was inhibited by venetoclax. Taken together, our findings suggest that although venetoclax promotes ROS accumulation and accelerates apoptosis in AML cells when combined with DMF, single-agent venetoclax does not compromise major antioxidant responses, including Nrf2 activation and GSH production.

### The combination of venetoclax and DMF synergistically impairs mitochondrial respiration and mitochondrial membrane potential in AML cell lines

**Fig. 4:**
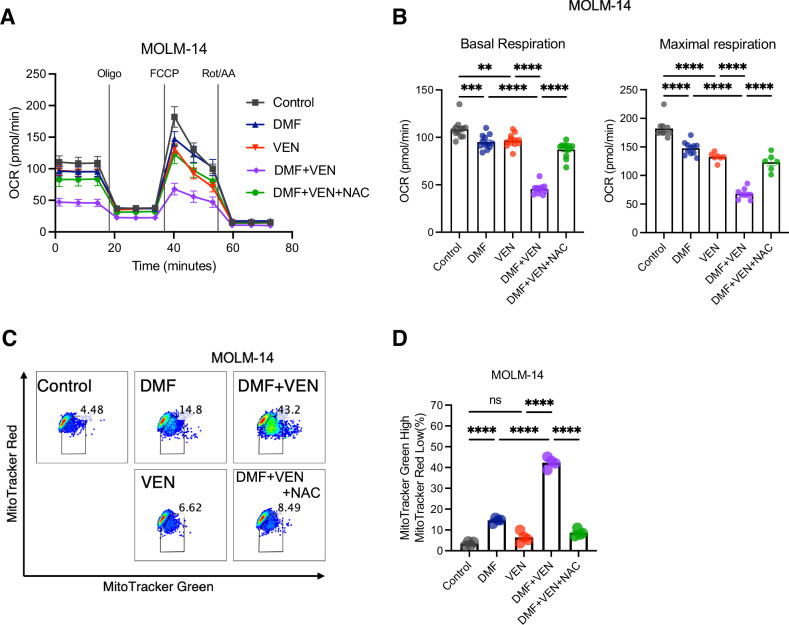
The combination of venetoclax and DMF synergistically disrupts mitochondrial integrity in AML cell lines. **A** Seahorse extracellular flux assay. OCR in MOLM-14 was measured after sequential treatment with Oligo, FCCP, and Rot/AA in MOLM-14 cells. Cells were preincubated with medium containing 0.1% DMSO (control), or DMF (50 µM) in the presence or absence of NAC (2.5 mM) overnight. Basal respiration was measured prior to sequential treatment. Graphs compare basal OCR and maximal OCR (*n* = 12 per group). Data from three independent experiments. **B** Bars show the basic respiration and maximal respiration rate under the indicated conditions. * *P* < 0.05, ** *P* < 0.01, *** *P* < 0.001, **** *P* < 0.0001, one-way ANOVA with Tukey’s post-hoc test. Shown as mean ± SEM. **C** Flow cytometric analysis of mitochondrial status on MOLM-14 measured by MitoTracker Green and MitoTracker Red. Data are representative of three independent experiments. **D** Bars show the comparison of MitoTracker Green high and MitoTracker Red low population (%) in MOLM-14 cells under the indicated conditions. **** *P* < 0.0001, ns: not significant, one-way ANOVA with Tukey’s post-hoc test.

**Fig. 5:**
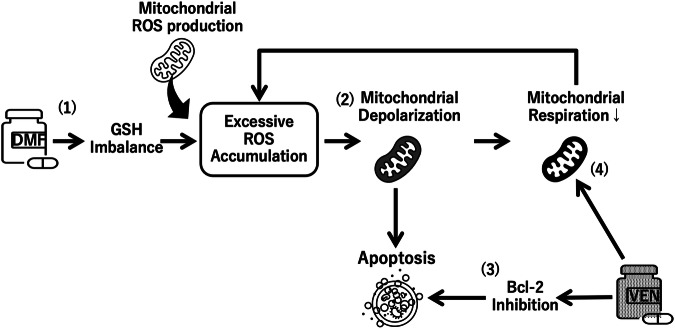
Proposed mechanism underlying the synergistic anti-leukemic effects of the combination of venetoclax and DMF in AML. (1) DMF inactivates the antioxidant GSH by cysteine succination, leading to ROS accumulation. (2) Excess ROS oxidize mitochondrial structures, triggering mitochondrial membrane depolarization and reducing ETC activity, eventually resulting in apoptotic cell death. (3) The BH3-mimetic venetoclax can accelerate DMF-induced apoptosis by inhibiting anti-apoptotic BCL-2 proteins (canonical pathway). (4) Venetoclax also inhibits mitochondrial respiration and TCA cycle activity (non-canonical pathway), further exacerbating ROS accumulation and creating a vicious cycle that amplifies cellular stress.

Although the increase in ROS induced by venetoclax appears to be small when used at low concentrations (Fig. 3G), we observed a dose-dependent rise in ROS levels with increasing venetoclax concentrations in MOLM-14 (Supplementary Fig. S4). We assume that venetoclax disrupts optimal mitochondrial respiration, leading to excess ROS generation, while this can be immediately mitigated by enhancing antioxidant machinery under normal conditions. Upon DMF treatment, however, oxidative stress may become evident due to GSH inactivation, thereby exacerbating ROS accumulation. Supporting this view, venetoclax reduced mitochondrial OXPHOS, and its combination with DMF led to further reduction in MOLM-14 cells (Fig. 4A, B). This reduction was significantly restored by NAC, but not to the basal level. Since the ETC takes place in the inner mitochondrial membrane, a loss of MMP immediately leads to inefficient ETC function, resulting in impaired OXPHOS activity. Indeed, venetoclax triggered mitochondrial depolarization, albeit modestly, which is most likely caused by BAX/BAK-mediated outer membrane permeabilization (Fig. 4C, D) [24]. When venetoclax was combined with DMF, the percentage of depolarized mitochondria was dramatically increased than with either agent alone. This synergy was again negated by NAC treatment. These observations suggest that DMF may have a priming effect on venetoclax-induced mitochondrial membrane damage through ROS accumulation, which in turn compromises mitochondrial integrity and eventually promotes apoptosis of AML cells. Figure 5 demonstrates the proposed mechanism underlying the anti-leukemic synergistic effects of the combination of venetoclax and DMF.

## Discussion

Recent evidence indicates that certain TCA cycle metabolites including fumarate play a pivotal role in regulating intracellular signaling and metabolic pathways, mainly through post-translational modification [25–27]. This study demonstrated that DMF inhibits GSH activity, leading to ROS accumulation, resulting in apoptotic cell death in AML cell lines. Elevated ROS levels are strongly correlated with reduced cell viability, and are also linked to reduced MMP and OXPHOS. Furthermore, we found a strong synergy between venetoclax and DMF: this combination led to higher ROS accumulation, reducing MMP, and eventually resulted in more apoptosis in AML cell lines compared to either single agent alone.

While the antitumor effects of DMF have been extensively investigated in solid tumors and lymphomas, there have been few reports about myeloid malignancies. Regarding lymphomas, DMF has been shown to induce apoptosis by suppressing nuclear factor kappa B (NF-κB) pathway in cutaneous T-cell lymphoma (CTCL) [28]. In diffuse large B-cell lymphoma, on the other hand, the mechanism of action by which DMF promotes apoptosis appears to differ between germinal center B-cell (GCB)-like and activated B-cell (ABC)-like subtypes [17]. Similar to the results observed in CTCL, the suppression of NF-κB appears to play an essential role in inducing apoptosis in the ABC DLBCL subtype, whereas the GCB DLBCL subtype exhibited lower GSH pool and glutathione peroxidase 4 levels, undergoing ferroptosis after DMF treatment. The authors reasoned that this difference can be attributed to differences in the expression of lipoxygenases (LOX), which are responsible for regulating the cellular pool of lipid hydroperoxides that trigger the initiation of ferroptosis: 5-LOX was highly expressed in GCB DLBCL, but was nearly absent in ABC DLBCL and myeloid cells. More importantly, however, we found that DMF promotes Nrf2 activation/nuclear translocation in AML cell lines, an effect that is theoretically conceivable given its antioxidant function, although this response is not sufficient to fully restore the redox balance. Nrf2 has been shown to inhibit the key process of ferroptosis by negatively regulating the genes associated with the capacity of labile iron pools and lipid peroxides [29]. This means that Nrf2 activation triggered by DMF may allow AML cells to circumvent ferroptosis but fails to prevent apoptosis caused by oxidative stress.

Consistent with our findings, studies have shown that suppressing antioxidant defense systems renders cancer cells vulnerable to apoptotic cell death induced by agents that further elevate ROS levels [1, 2]. Oxidative damage to mitochondria components can exacerbate ROS accumulation due to ETC dysfunction, creating a vicious cycle that amplifies cellular stress through ROS-induced ROS release [30]. Venetoclax has been shown to induce apoptosis of cancer cells not only through its canonical pathway, but also by impairing mitochondrial metabolism and ETC activity (non-canonical pathway) [18, 31, 32]. A recent report has shown that venetoclax inhibits mitochondrial respiration and TCA cycle activity in colon cancer cells that are deficient in BCL-2 or BAX/BAK expression [31]. Unlike venetoclax treatment, targeting deletion of BCL-2 gene had no effect on mitochondrial OXPHOS [31], suggesting that this mechanism is independent of BCL-2, although there is still some controversy between studies [18]. In this study, we did not find a significant difference in ROS levels in venetoclax-refractory BCL-2-low U937 cells following venetoclax treatment. However, a marked increase in cellular ROS and apoptosis was observed when DMF was used concomitantly, suggesting that DMF may boost venetoclax-mediated mitochondrial dysfunction. NAC or Z-VAD-fmk restored cell viability in AML cells, indicating that ROS-dependent caspase activation is the underlying cause of this synergistic induction of apoptosis. Another report demonstrated that while single-agent venetoclax did induce ROS accumulation in leukemia stem cells from patients, it had no effect on mitochondrial respiration. However, venetoclax in combination with azacitidine led to significant reductions in OXPHOS, supporting a combined therapeutic strategy targeting the redox balance, similar to ours [32].

In summary, our study proposes a novel combination therapy for AML that uses orally available agents, venetoclax and DMF, both of which have been used worldwide and have been proven to be well-tolerated with manageable toxicities. One potential concern is an increased risk of infection, as grade 2 or higher lymphopenia has been reported in nearly 30 to 40% of multiple sclerosis patients receiving DMF treatment [33, 34]. Additionally, this study did not clarify the biological characteristics that determine the susceptibility of AML cells to oxidative cellular damage, which may be another limitation. Given that apoptosis is the primary mode of cell death induced by DMF through ROS accumulation, p53 status may be a key determinant of drug response; MOLM-14 expresses wild-type p53, whereas KG-1a harbors a mutated form, and THP-1 and U937 are p53-null (Supplementary Table S3). Moreover, MOLM-14 exhibits high OXPHOS activity, along with increased mitochondrial mass and ROS levels, whereas KG-1a and, particularly, U937 cells display significantly lower bioenergetic status [35]. These biological and genetic differences may contribute to varying degrees of DMF sensitivity among leukemia cells. Further studies in animal models are needed to confirm the safety and efficacy of this treatment, specifically identifying which leukemia subtypes would benefit the most from this combination strategy.

## Supplementary information

Supplementary Figure S1

Supplementary Figure S2

Supplementary Figure S3

Supplementary Figure S4

Figure legend of Supplementary Figure S1 to 4

Supplementary Table S1

Supplementary Table S2

Supplementary Table S3

The raw data of Western Blot: TBP

The raw data of Western Blot: Nrf2

The raw data of Western Blot: B-actin

## Data Availability

The data generated and/or analysed during the current study will be provided by the corresponding author on reasonable request.
