# Supplementary figures and images for: The combination of venetoclax with dimethyl fumarate synergistically induces apoptosis in AML cells by disrupting mitochondrial integrity through ROS accumulation
Source: Cell Death Dis. 2025 Oct 21;16(1):750. doi: 10.1038/s41419-025-08040-x (PMC12541053; doi:10.1038/s41419-025-08040-x)

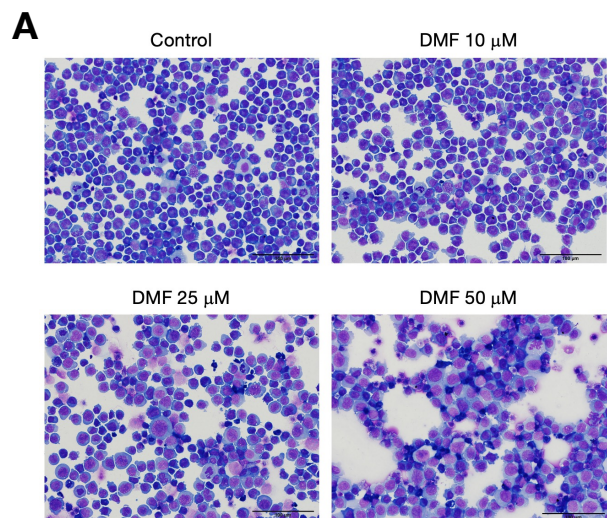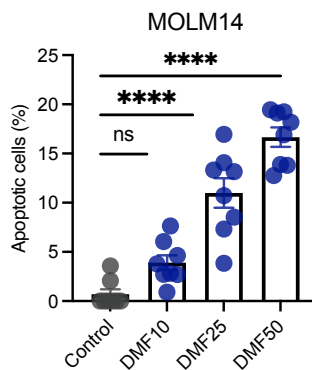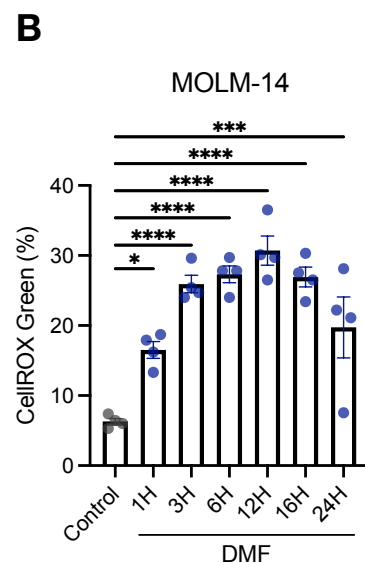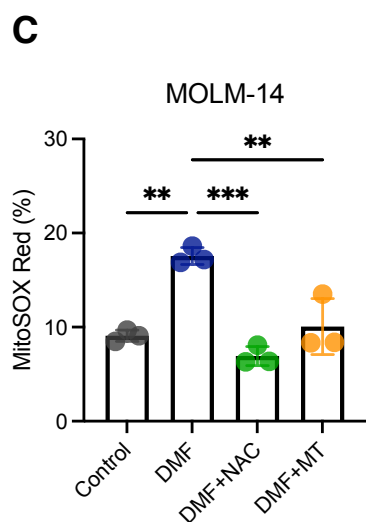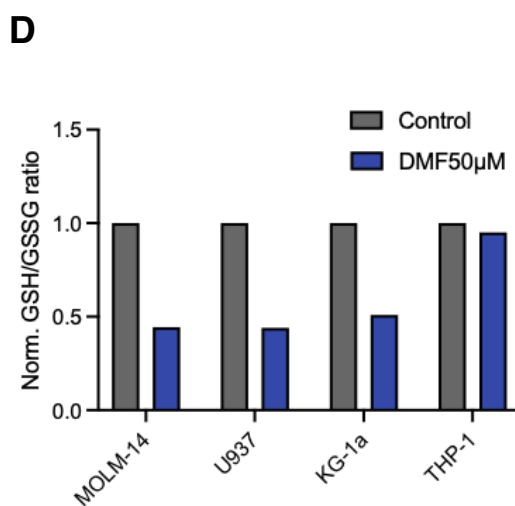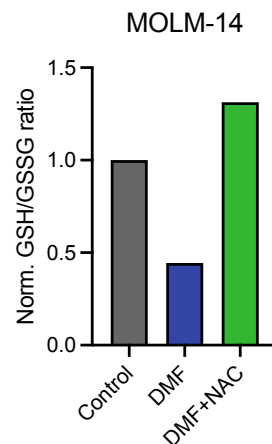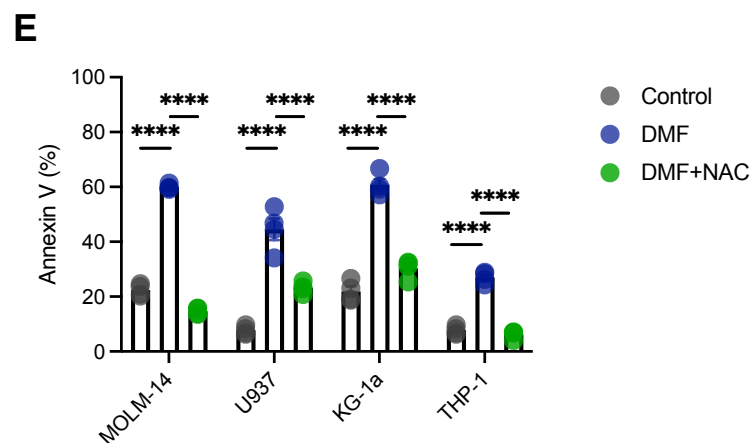

Supplement: Supplementary file 1 — Supplementary Figure S1 [file 41419_2025_8040_MOESM1_ESM.pdf]

MOLM-14

DMF

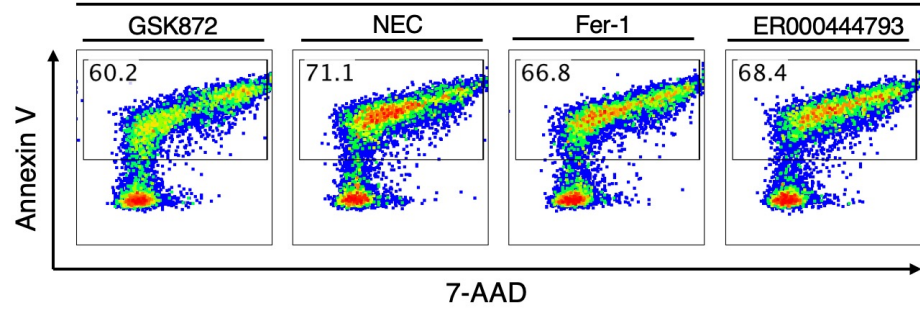

Supplement: Supplementary file 2 — Supplementary Figure S2 [file 41419_2025_8040_MOESM2_ESM.pdf]

## A

### MOLM-14

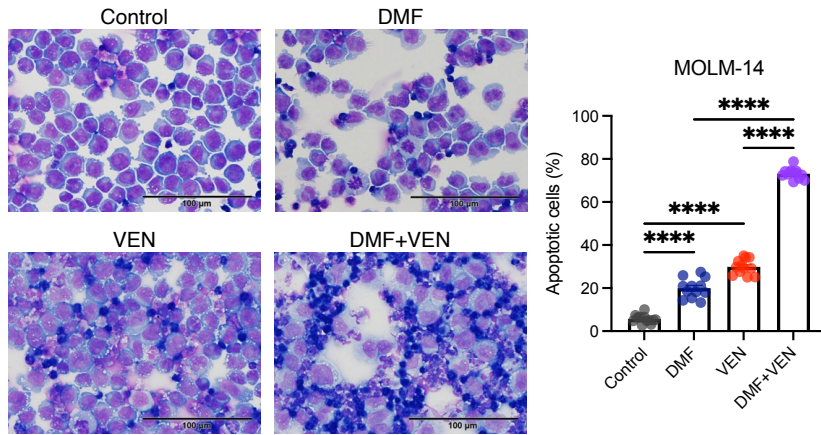

## B

### human CD34

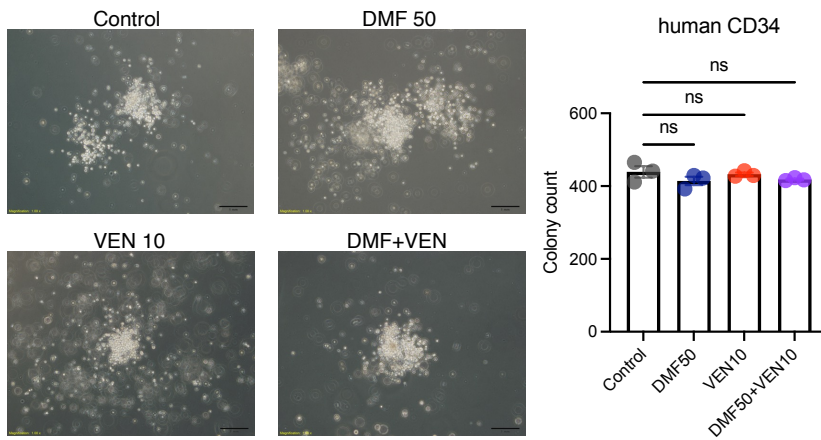

## C

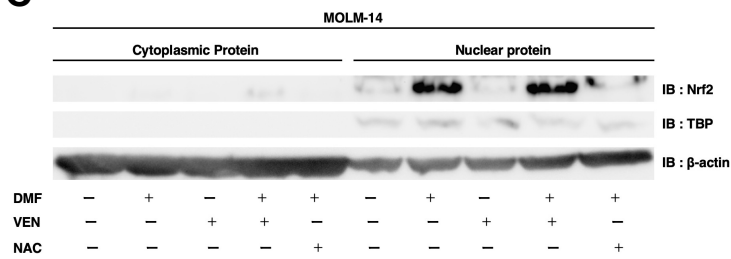

## D

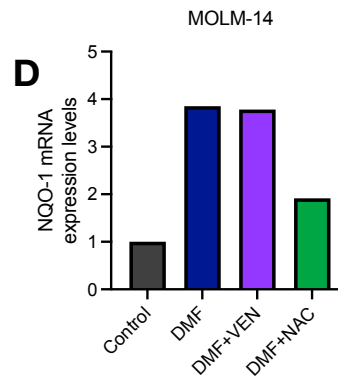

## E

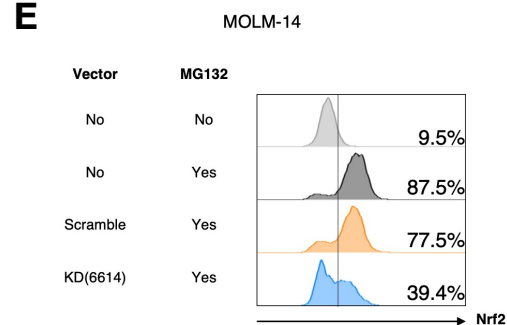

## F

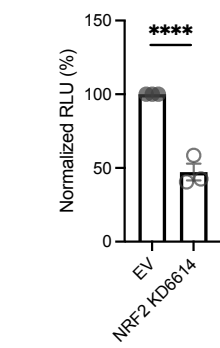

## G

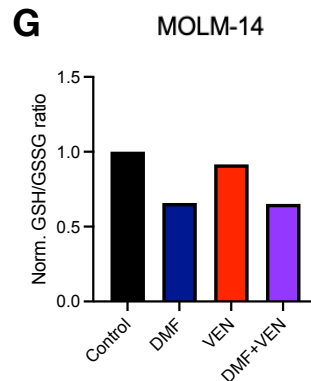

## H

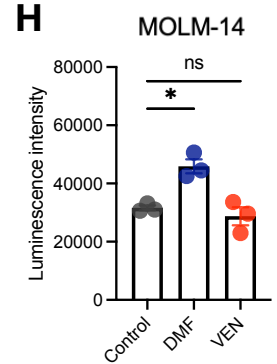

Supplement: Supplementary file 3 — Supplementary Figure S3 [file 41419_2025_8040_MOESM3_ESM.pdf]

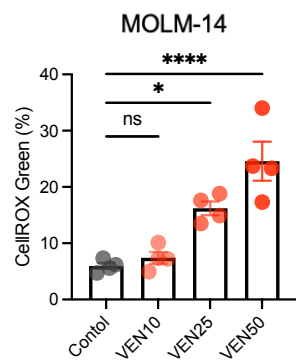

Supplement: Supplementary file 4 — Supplementary Figure S4 [file 41419_2025_8040_MOESM4_ESM.pdf]

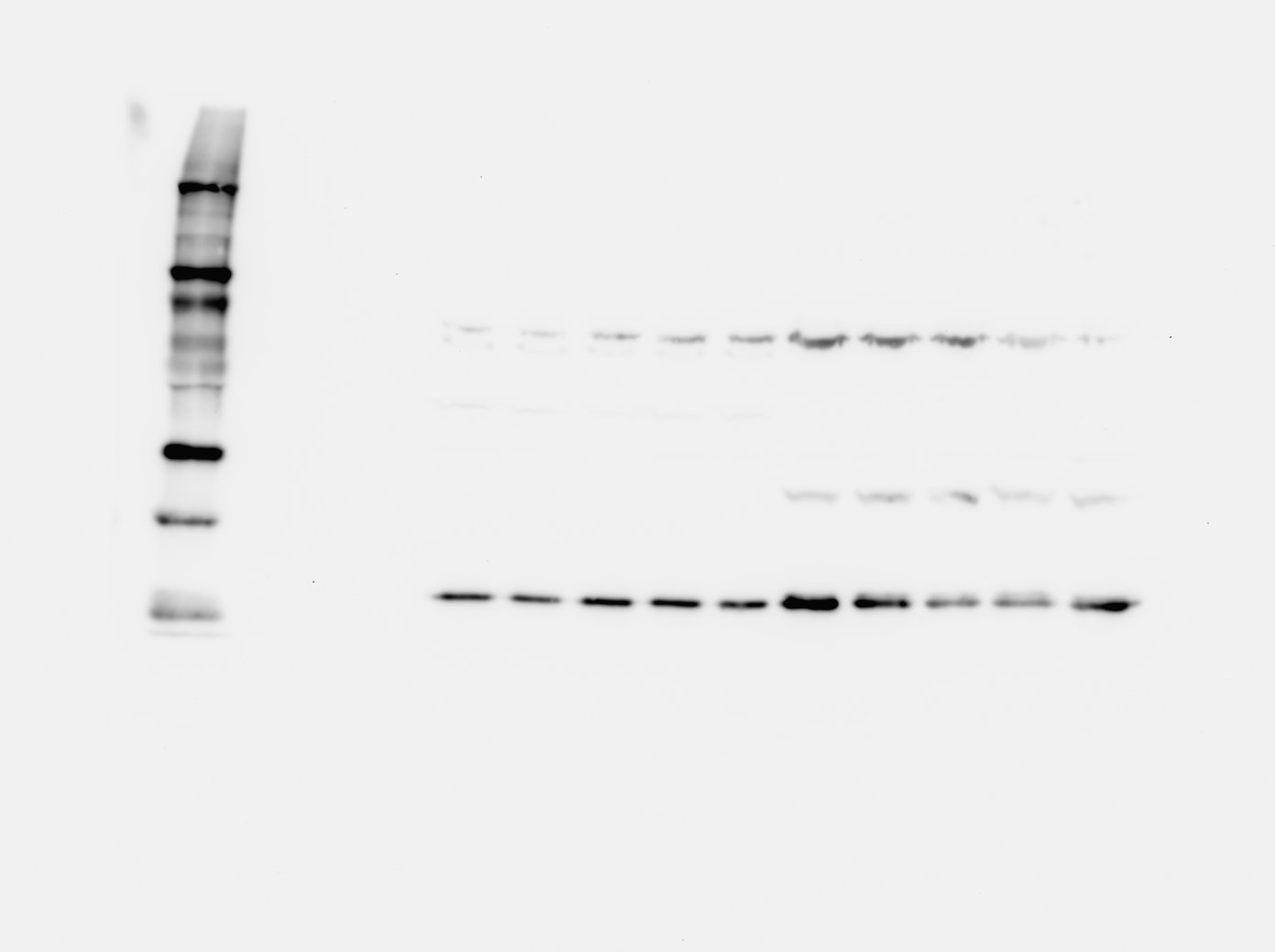

Supplement: Supplementary file 9 — The raw data of Western Blot: TBP [file 41419_2025_8040_MOESM9_ESM.png]

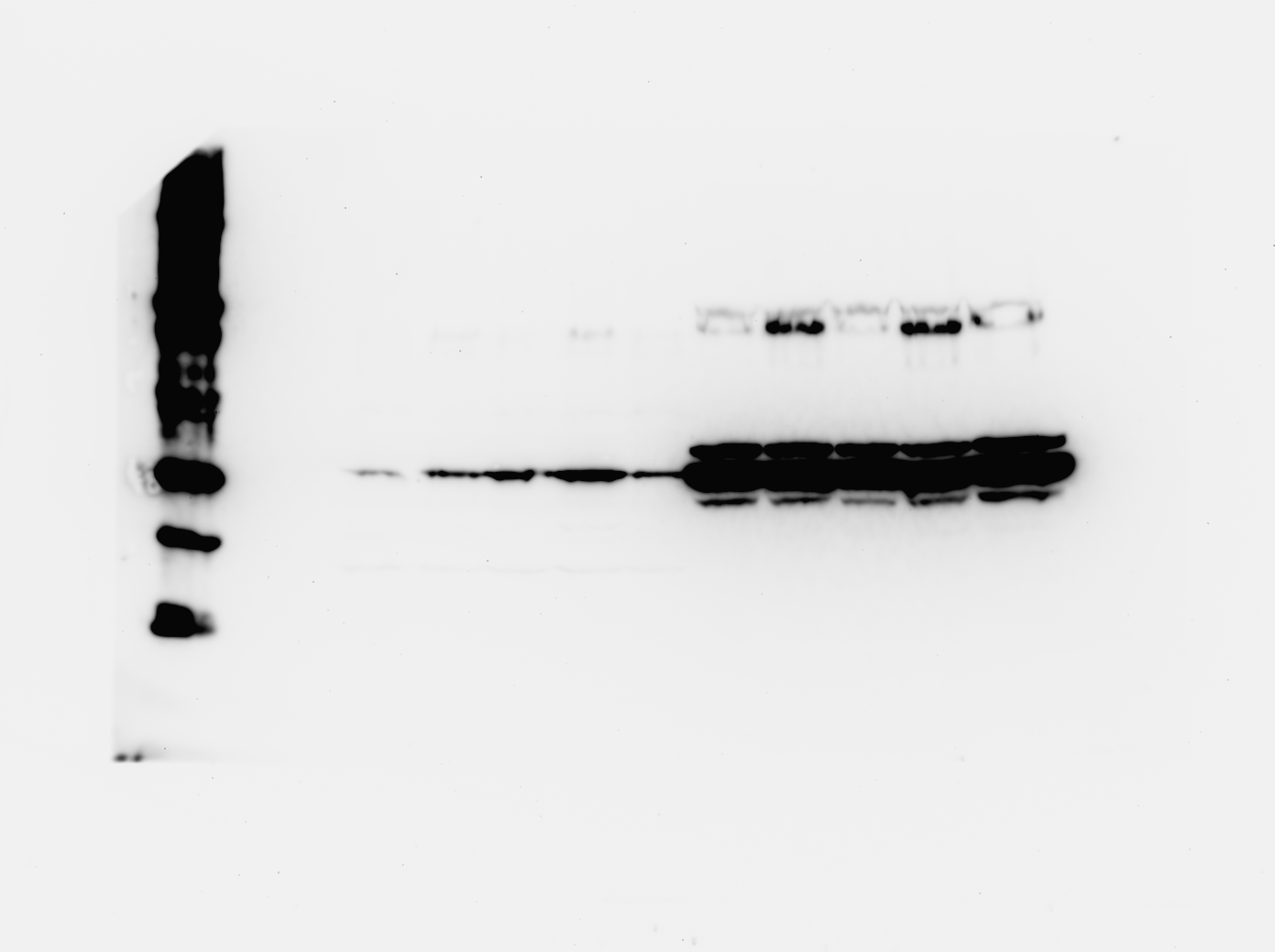

Supplement: Supplementary file 10 — The raw data of Western Blot: Nrf2 [file 41419_2025_8040_MOESM10_ESM.png]

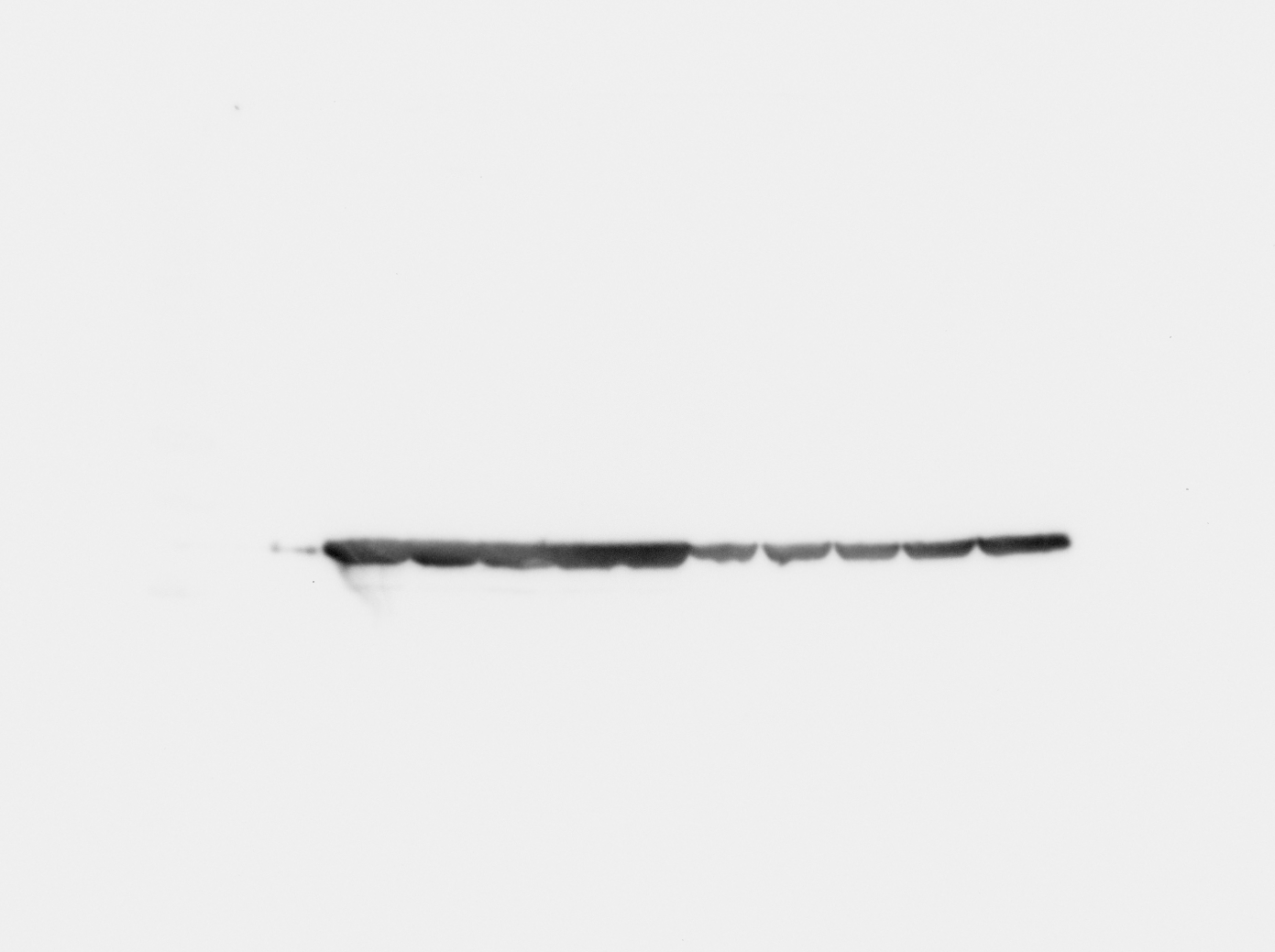

Supplement: Supplementary file 11 — The raw data of Western Blot: B-actin [file 41419_2025_8040_MOESM11_ESM.png]
