# Supplementary material for: The combination of venetoclax with dimethyl fumarate synergistically induces apoptosis in AML cells by disrupting mitochondrial integrity through ROS accumulation
Source: Cell Death Dis. 2025 Oct 21;16(1):750. doi: 10.1038/s41419-025-08040-x (PMC12541053; doi:10.1038/s41419-025-08040-x)
Supplement: Supplementary file 5 — Figure legend of Supplementary Figure S1 to 4 [file 41419_2025_8040_MOESM5_ESM.docx]

**Supplementary Fig. S1**

1. Representative optical microscopical images of MOLM-14 cells (20x). Bar graph showing morphologically apoptotic cells (%). **** *P* < 0.0001, ns: not significant, one-way ANOVA with Tukey’s post-hoc test. Shown as mean ± SEM (n = 8 per group).
2. Time-course analysis of apoptosis in MOLM-14 cells treated with DMF. * *P* < 0.05, *** *P* < 0.001, **** *P* < 0.0001, one-way ANOVA with Tukey’s post-hoc test. Shown as mean ± SEM (n = 4 per group).Cells were treated with DMF (50µM) and analyzed at the indicated time points.
3. Mitochondrial ROS levels determined by MitoSOX Red using flow cytometry. ** *P* < 0.01, *** *P* < 0.001, one-way ANOVA with Tukey’s post-hoc test. Shown as mean ± SEM (n=3 per group).
4. Ratio of total reduced glutathione (GSH) to oxidized glutathione (GSSH) in AML cell lines treated with DMF (50 μM) overnight. NAC was used at 2.5 mM. The values were normalized with respect to the control group (0.1% DMSO). Representative data from one of three independent experiments.
5. Annexin V-positive cells (%) in human AML cell lines. DMF was used at concentrations of 50 µM for MOLM-14, 100 µM for KG-1a, and 300 µM for U937 and THP-1, respectively. NAC was used at 2.5 mM. **** *P* < 0.0001, one-way ANOVA with Tukey’s post-hoc test. Shown as mean ± SEM (n = 4 per group)

**Supplementary Fig. S2**

Representative dot plots of Annexin V and 7-AAD on MOLM-14 cells from one of five experiments. Cells (2 × 10^5^ cells/well in a 12-well plate) were exposed to medium containing DMF (50 µM) in the presence of the indicated inhibitors (GSK872 (10 μM), Necrostatin-1 (NEC, 30 μM), Ferrostatin-1 (Fer-1, 5 μM), or ER000444793 (ER, 10 μM)) for 24 hours.

**Supplementary Fig. S3**

1. Representative optical microscopical images of MOLM-14 cells treated with the combination of venetoclax (10 nM) and DMF (50 μM) for 24 hours (20x). Scale bars 100μm. Bar graph showing morphologically apoptotic cells (%). **** *P* < 0.0001, one-way ANOVA with Tukey’s post-hoc test. Shown as mean ± SEM (n = 12 per group).
2. Colony-forming unit assay for human normal CD34^+^ cells (x 40) cultured in semi-solid methylcellulose-based medium with DMF (50 μM), venetoclax (10 nM), or the combination of venetoclax and DMF. Scale bars 1mm. Bar graph showing morphologically apoptotic cells (%). **** *P* < 0.0001, one-way ANOVA with Tukey’s post-hoc test. Shown as mean ± SEM (n = 3 per group).
3. Western blot analysis of Nrf2, TBP, and β-actin on MOLM-14 cells taken 4 hours after incubation with the indicated concentrations of agents.
4. NQO1 mRNA expression levels normalized to the control in MOLM14 cells. Cells were treated under the indicated conditions for 6 hours. Data from three independent experiments.
5. Comparison of Nrf2 expression by flow cytometry. MG132, a proteasome inhibitor, was used at 10 μM for 2 hours.
6. Relative antioxidant response element (ARE) reporter luciferase activity in wild-type MOLM-14 and Nrf2-knocked down (KD) MOLM-14 cells.
7. Normalized GSH/GSSG. MOLM-14 cells were extracted and analyzed after treatment with DMF (50 µM) or in combination with venetoclax (10 nM) for 24 hours. The values were normalized with respect to the control group (0.1% DMSO). Representative data from one of three independent experiments.
8. Cystine uptake measured by a fluorescence-based assay (Cystine Uptake Assay Kit, DOJINDO). * *P* < 0.05, ns: not significant, one-way ANOVA with Tukey’s post-hoc test. Shown as mean ± SEM (n = 3 per group).

**Supplementary Fig. S4**

Cellular ROS levels in MOLM-14 determined by CellROX Green using flow cytometry. * *P* < 0.05, **** *P* < 0.0001, ns: not significant, one-way ANOVA with Tukey’s post-hoc test. Shown as mean ± SEM.
