# Supplementary material for: The combination of venetoclax with dimethyl fumarate synergistically induces apoptosis in AML cells by disrupting mitochondrial integrity through ROS accumulation
Source: Cell Death Dis. 2025 Oct 21;16(1):750. doi: 10.1038/s41419-025-08040-x (PMC12541053; doi:10.1038/s41419-025-08040-x)
Supplement: Supplementary file 6 — Supplementary Table S1 [file 41419_2025_8040_MOESM6_ESM.pptx]

## Slide 1
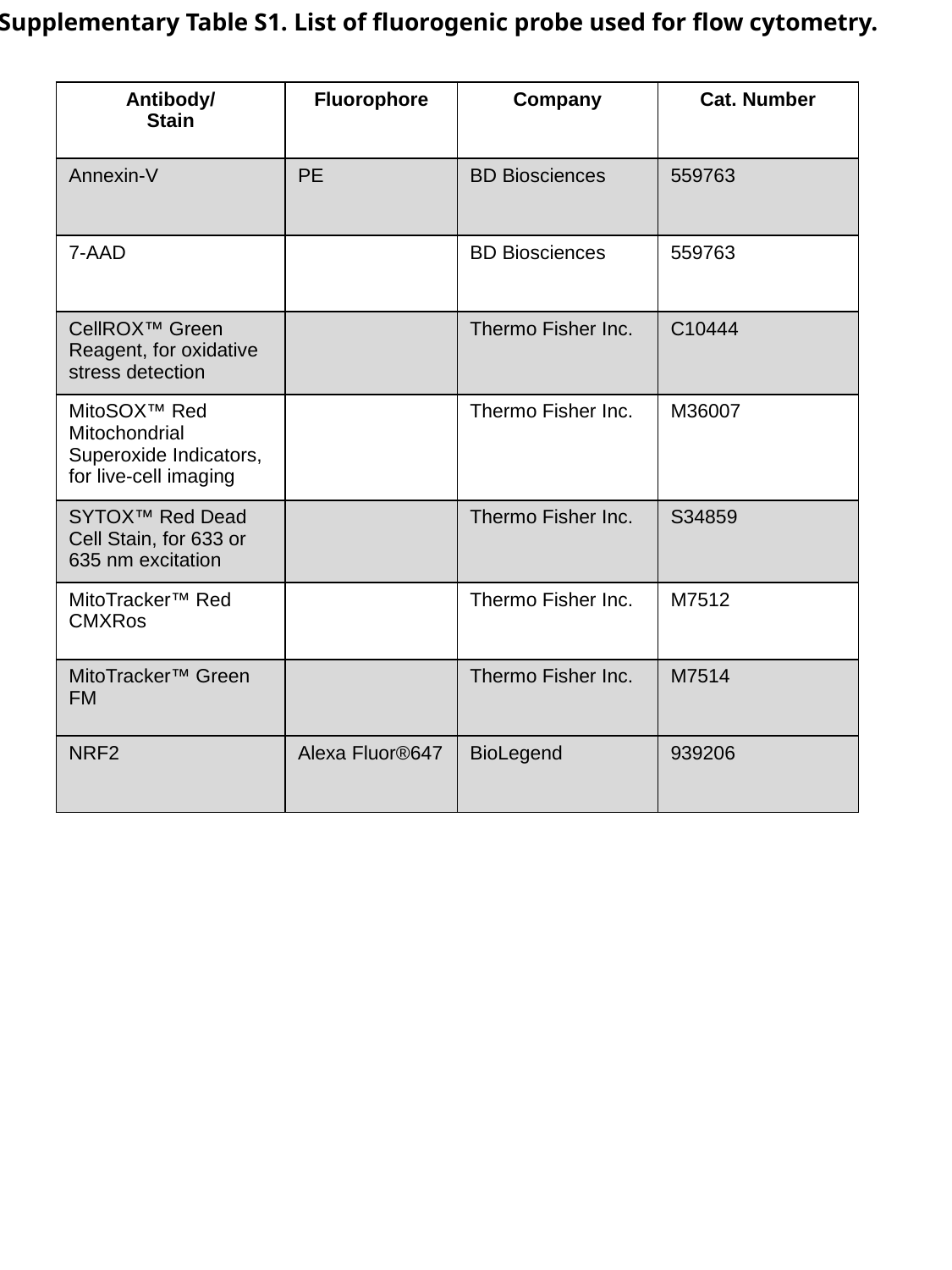

Supplementary Table S1. List of fluorogenic probe used for flow cytometry.
| Antibody/ Stain | Fluorophore | Company | Cat. Number |
| --- | --- | --- | --- |
| Annexin-V | PE | BD Biosciences | 559763 |
| 7-AAD | | BD Biosciences | 559763 |
| CellROX™ Green Reagent, for oxidative stress detection | | Thermo Fisher Inc. | C10444 |
| MitoSOX™ Red Mitochondrial Superoxide Indicators, for live-cell imaging | | Thermo Fisher Inc. | M36007 |
| SYTOX™ Red Dead Cell Stain, for 633 or 635 nm excitation | | Thermo Fisher Inc. | S34859 |
| MitoTracker™ Red CMXRos | | Thermo Fisher Inc. | M7512 |
| MitoTracker™ Green FM | | Thermo Fisher Inc. | M7514 |
| NRF2 | Alexa Fluor®647 | BioLegend | 939206 |
