# Supplementary material for: The combination of venetoclax with dimethyl fumarate synergistically induces apoptosis in AML cells by disrupting mitochondrial integrity through ROS accumulation
Source: Cell Death Dis. 2025 Oct 21;16(1):750. doi: 10.1038/s41419-025-08040-x (PMC12541053; doi:10.1038/s41419-025-08040-x)
Supplement: Supplementary file 7 — Supplementary Table S2 [file 41419_2025_8040_MOESM7_ESM.pptx]

## Slide 1
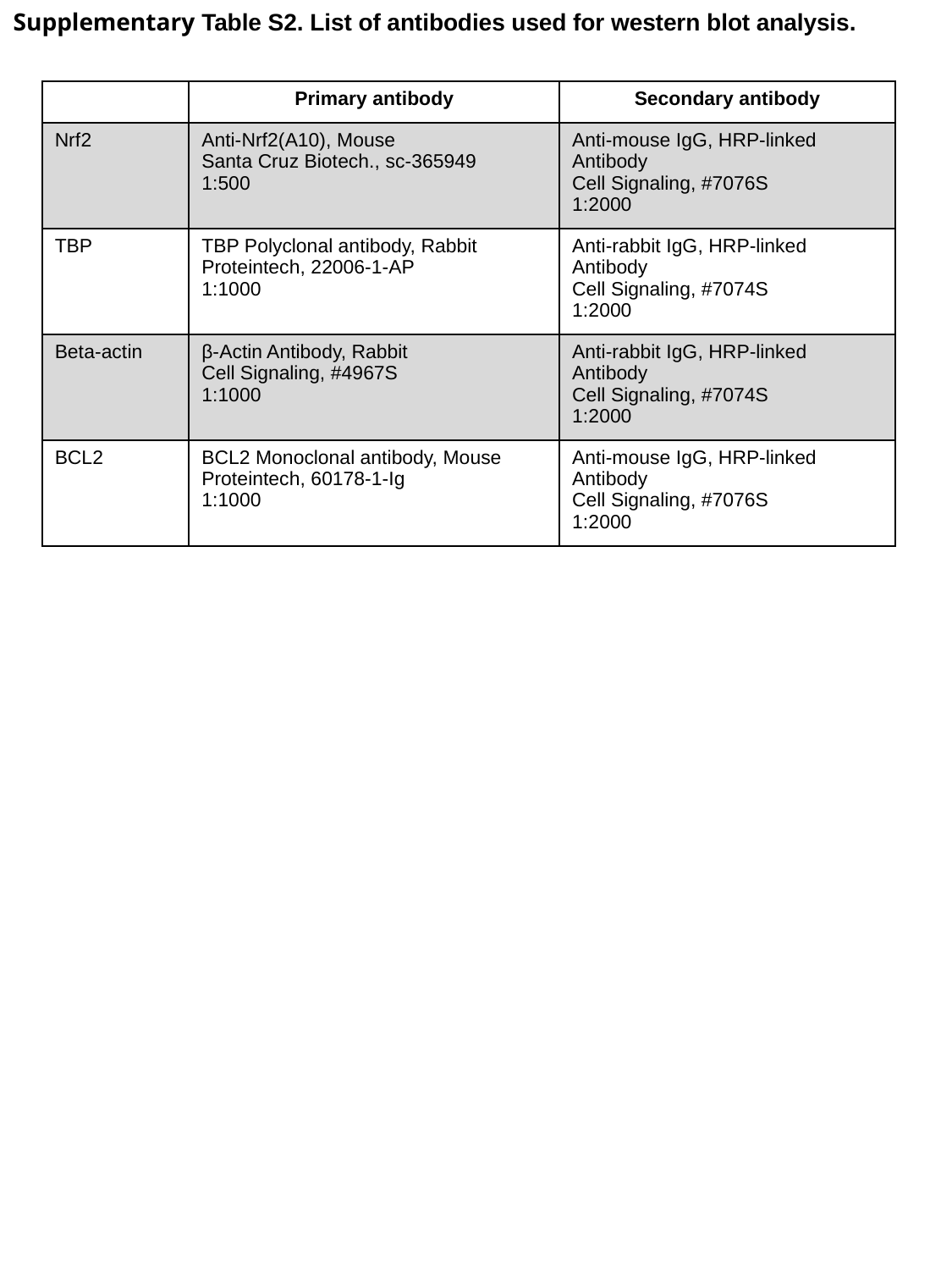

Supplementary Table S2. List of antibodies used for western blot analysis.
| | Primary antibody | Secondary antibody |
| --- | --- | --- |
| Nrf2 | Anti-Nrf2(A10), Mouse Santa Cruz Biotech., sc-365949 1:500 | Anti-mouse IgG, HRP-linked Antibody Cell Signaling, #7076S 1:2000 |
| TBP | TBP Polyclonal antibody, Rabbit Proteintech, 22006-1-AP 1:1000 | Anti-rabbit IgG, HRP-linked Antibody Cell Signaling, #7074S 1:2000 |
| Beta-actin | β-Actin Antibody, Rabbit Cell Signaling, #4967S 1:1000 | Anti-rabbit IgG, HRP-linked Antibody Cell Signaling, #7074S 1:2000 |
| BCL2 | BCL2 Monoclonal antibody, Mouse Proteintech, 60178-1-Ig 1:1000 | Anti-mouse IgG, HRP-linked Antibody Cell Signaling, #7076S 1:2000 |
