# Supplementary material for: The combination of venetoclax with dimethyl fumarate synergistically induces apoptosis in AML cells by disrupting mitochondrial integrity through ROS accumulation
Source: Cell Death Dis. 2025 Oct 21;16(1):750. doi: 10.1038/s41419-025-08040-x (PMC12541053; doi:10.1038/s41419-025-08040-x)
Supplement: Supplementary file 8 — Supplementary Table S3 [file 41419_2025_8040_MOESM8_ESM.pptx]

## Slide 1
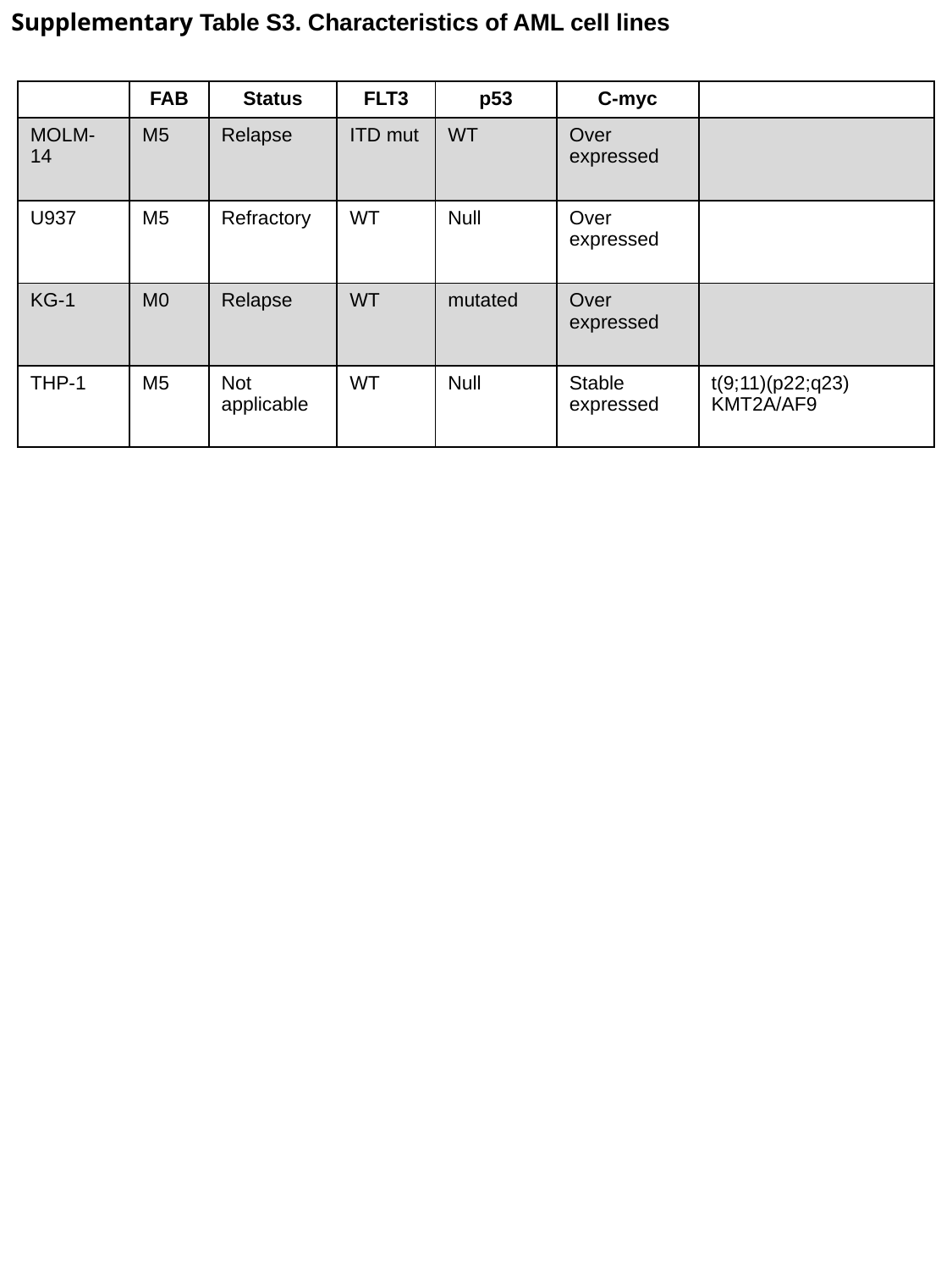

Supplementary Table S3. Characteristics of AML cell lines
| | FAB | Status | FLT3 | p53 | C-myc | |
| --- | --- | --- | --- | --- | --- | --- |
| MOLM-14 | M5 | Relapse | ITD mut | WT | Over expressed | |
| U937 | M5 | Refractory | WT | Null | Over expressed | |
| KG-1 | M0 | Relapse | WT | mutated | Over expressed | |
| THP-1 | M5 | Not applicable | WT | Null | Stable expressed | t(9;11)(p22;q23) KMT2A/AF9 |
